# Action and Valence Modulate Choice and Choice-Induced Preference Change

**DOI:** 10.1371/journal.pone.0119682

**Published:** 2015-03-06

**Authors:** Raphael Koster, Emrah Duzel, Raymond J. Dolan

**Affiliations:** 1 Institute of Cognitive Neuroscience, University College London, London, United Kingdom; 2 Wellcome Trust Centre for Neuroimaging, Institute of Neurology, University College London, London, United Kingdom; 3 Otto von Guericke University Magdeburg, Institute of Cognitive Neurology and Dementia Research, Magdeburg, Germany; 4 German Center for Neurodegenerative Diseases, Magdeburg, Germany; Brain and Spine Institute (ICM), FRANCE

## Abstract

Choices are not only communicated via explicit actions but also passively through inaction. In this study we investigated how active or passive choice impacts upon the choice process itself as well as a preference change induced by choice. Subjects were tasked to select a preference for unfamiliar photographs by action or inaction, before and after they gave valuation ratings for all photographs. We replicate a finding that valuation increases for chosen items and decreases for unchosen items compared to a control condition in which the choice was made post re-evaluation. Whether choice was expressed actively or passively affected the dynamics of revaluation differently for positive and negatively valenced items. Additionally, the choice itself was biased towards action such that subjects tended to choose a photograph obtained by action more often than a photographed obtained through inaction. These results highlight intrinsic biases consistent with a tight coupling of action and reward and add to an emerging understanding of how the mode of action itself, and not just an associated outcome, modulates the decision making process.

## Introduction

Choosing according to our preferences is an everyday occurrence in our lives. Some choices are expressed explicitly through action while others are expressed passively by accepting a default or not altering an existing choice. Interestingly, an xkcd comic titled “Regrets” (http://xkcd.com/458/) points out that there are more Google hits for “I should have kissed him/her” compared to “I shouldn’t have kissed him/her”. This asymmetry of regret for action and inaction has been linked to a number of possible explanations and mediating factors including sense of agency [1], cognitive accessibility [2], and differential mutability [3,4]. Another possible explanation for this effect is that the consequences of actions are revaluated more easily than the consequences of inaction. In this study we ask how different modes of choice expression, i.e. action and inaction, shape the choice process and the following choice-induced preference change in different valence contexts.

The observation that choices express, but also shape our preferences is a consistent finding [5]. One paradigm demonstrating how choice affects preferences is the free-choice paradigm wherein value judgements are gathered on options before and after subjects are forced to choose one and reject the other option [6,7]. Post choice the chosen option is valued higher than the unchosen option, a spread seen for hard choices (options the subject initially valued similarly) but not easy choices (options which the subject initially valued differently). A classical explanation of this finding is that it reflects a reduction of cognitive dissonance [8] where attitudes are changed to evaluate the current reality more positively. A recent study has highlighted a methodological flaw in the way many studies on choice-induced preference change have been conducted [6]. As all valuations contain noise, chosen items are less likely to decrease in a second valuation because the choice accesses the same true underlying value. This is strikingly revealed by a control condition in which a choice is made after the second evaluation, ruling out any causal influence of the choice on the change in valuation (see Izuma & Murayama [7] for review). However, despite this flaw in the literature recent studies replicated choice-induced preference change with experimental designs that account for this artefact [9–13].

There are number of reasons to hypothesize that choice and choice-induced preference change are modulated by action. A recent study [14] reported that the relative value of visually represented items is enhanced by button presses to a coinciding auditory cue. This ‘cue-approach training’ increased choice preference for otherwise equally valued items and led to a subsequently increased valuation. Interestingly, this effect was only seen for items with relatively high value. Additionally, a revaluation effect was driven by a value change induced by the preceding choice, indicating a specificity of the cue approach training to bias the binary choice between two items. The observation that the effect is only present for high value items points to a specific interaction of approach with choice and positive value. We believe that this is consistent with a Pavlovian congruence framework [15], in which button presses are a model for approach behaviour. Indeed, a coupling of action and reward could affect choice-induced preference change as it has been linked to dopaminergic circuitry [16,17] and an effect in the striatum [10,18]. Therefore, we hypothesize a value enhancing effect by action in a context of positive value.

**Fig 1:**
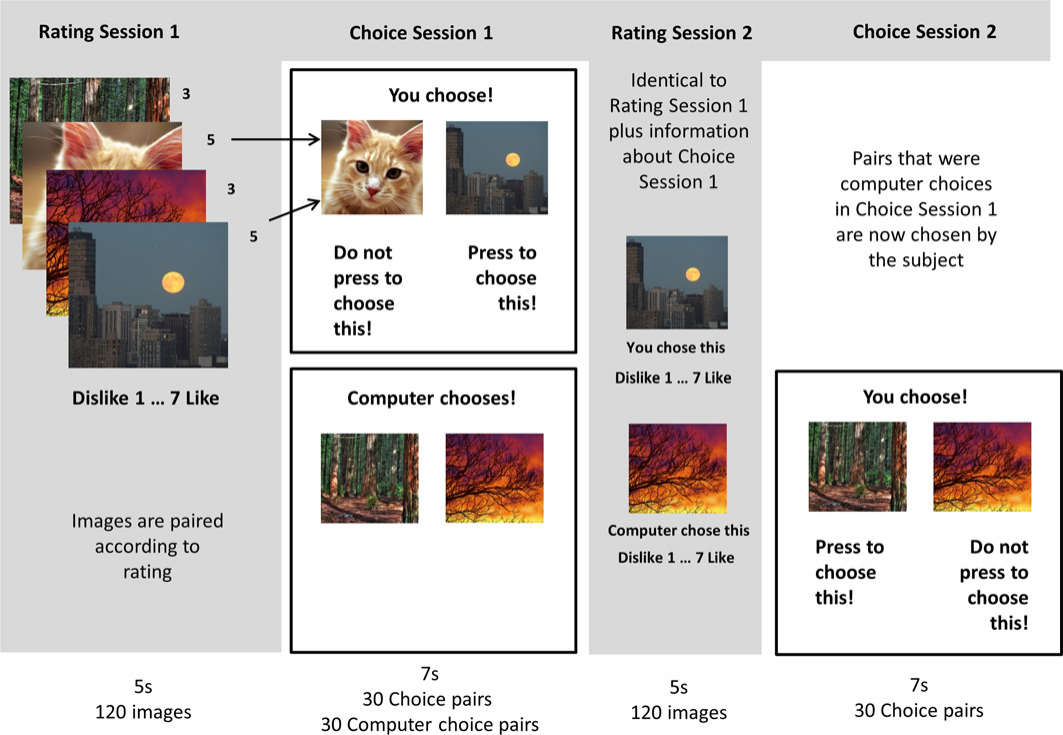
Experimental Design. The experimental paradigm comprised four distinct sessions. In Rating Session 1 subjects rated all images. These images were then paired on the basis of matched ratings. In Choice Session 1 subjects could freely choose (by either pressing or not pressing a button) or a computer chose at random. In Rating Session 2 subject rated all images again while text informed subjects as to what occurred in Choice Session 1. Finally, in Choice Session 2 the pairs the computer chose beforehand in Choice Session 1 were displayed. Subjects freely chose again by performing or omitting a button press like in Choice Session 1. This experiment was conducted in two independent samples with two different stimulus sets (positive and negative valence). Comparing the value change from Rating Session 1 to 2 for chosen and unchosen items before revaluation and after revaluation provided a measure of choice-induced value change. We tested whether this choice-induced value change was modulated by action or inaction (Go/No Go) and by valence. The frequency of choice expressed in a response Go or No Go allows us to test for a bias induced by action on choice itself.

As described in Fig. 1, we adapted a free choice paradigm [9,10,19] to investigate the impact of approach on choice and choice-induced preference-change. Choice-induced value change is measured as the rating change between sessions for each item. The difference in this rating change for chosen and unchosen items (chosen-unchosen spread) reflects choice but also contains noise [6,7]. To calculate the genuine choice-induced preference change the chosen-unchosen spread is compared between the two experimental conditions, namely the experimental condition in which choice precedes revaluation and the control condition in which the choice is made after revaluation [6,7]. Crucially, choice itself was expressed either by action via button press (Go, as model for approach) or inaction (No Go), allowing us to test whether approach biases the choice itself and whether revaluation is modulated as a function of how the choice is expressed (Go or No Go). By conducting the experiment in two independent samples with a positive or negative valence stimulus set we could also test the hypothesis whether valence had a differential effect on a modulation of value and combined effect with action.

## Materials and Methods

### Subjects

Two groups (positive valence and negative valence stimuli) of 30 subjects participated in the study. Participants were recruited through a UCL participant pool, were self-reported right-handers, had normal or corrected to normal vision and were paid for their participation. The study was approved by the UCL local research ethics committee (PWB/ED/11–10–12b and 3450/002) and subjects gave informed written consent. To exclude subjects with poor compliance with task instructions subjects with more than 10 missed responses were removed from the analysis leaving 24 in the first group (4 males, mean age: 21.86) and 27 in the second group (12 males, mean age: 22.52).

### Experimental procedure

The two experimental groups only differed in the stimuli with which they were presented and what the outcome would be incentivising accurate choice. In the positive valence group, the pictures comprised diverse colour photographs gathered from various sources on the internet including landscapes and juvenile animals (note that the images in Fig. 1 were selected for being free of copy right and were not actually contained in the set used in the experiment). In the negative valence group images were selected from the IAPS [20] including dangerous animals, mutilations and depictions of violence.

The experimental paradigm contained four parts comprising a Rating Session 1, Choice Session 1, Rating Session 2 and Choice Session 2 (Fig. 1). In Rating Session 1 subjects were presented with 120 different images and asked to express how much they liked the picture on a scale from 1 to 7 with a button press. Subjects were instructed to use the whole range of the scale. Subjects had to respond within 5 seconds and the image was displayed on the screen for the full 5 seconds independent of the reaction time. The images were then paired to match liking rating, and randomly allocated to the “Session 1 Choice” or “Session 2 Choice” conditions. Note, that in previous studies [10,18] some pairs included two stimuli that were of unequal initial rating making the choice an “easy choice” (because one stimuli was clearly preferred over the other). No choice-induced preference change was expected for “easy choices” because the choice would not induce cognitive dissonance. Therefore, this condition was used previously as control condition. However, in the current study all pairs were matched for initial preference rating, rendering them all “hard choices” (or “critical pairs”). The “easy choice” control condition was omitted in the current experiment as the crucial control condition is choice after the second rating (rate-rate-choose) instead of before the second rating (rate-choose-rate; see Izuma & Murayama [7] for review). In Choice Session 1, pairs in the “Session 1 Choice” condition were displayed after the text “You choose!” was displayed on the screen for one second. One picture could be chosen by pressing the space bar (Go), the other picture could be chosen by not pressing anything (No Go). The side on which each picture was presented was allocated at random, as was which option was chosen by a Go or No Go response. Subjects had 7 seconds to make the choice and the pictures were displayed for the full 7 seconds independent of the time of choice. When subjects pressed the space bar word “chosen” would appear under the chosen option or if they did not press the space bar the word “chosen” would appear under the chosen option at the end of the choice period for 500ms. If the pair was in the “Session 2 Choice” condition, the message before the trials read “Computer chooses!” and subjects were instructed to observe the computer making a random choice. The choice the computer made was flagged by displaying the word “chosen” under the chosen option for 500ms. This condition allowed to keep exposure to images and choice constant and only manipulate whether subjects can attribute the choice to themselves. Note that the display time of each image was kept constant independent of subject’s behaviour in both conditions. A blank screen was displayed between trials for 500 ms. Trials were presented in random order. Rating Session 2 was identical to Rating Session 1 except that each picture was captioned with text stating whether the picture was chosen or not chosen by the subject or computer in Choice Session 1 (e.g. “You chose this.” for images that were chosen in the “Session 1 Choice” condition or “The computer rejected this.” for images that were rejected by the computer in the “Session 2 Choice” condition, see Izuma et al. [10] for a similar procedure). Note, that for the items in the “Session 1 Choice” condition subjects were informed about their own choice. This was not possible for items in “Session 2 Choice”, as subjects have not made a choice yet. Choice Session 2 presented all pairs in the “Session 2 Choice” condition for subjects to freely chose, identical to the pairs in the “Session 1 Choice” condition in Choice Session 1. In order to motivate subjects to make choices that reflect their preference, subjects in the positive valence group were told that in the end one of the choices is going to be chosen at random and implemented in reality and subjects did indeed receive a printout of a picture to take home. Subjects in the negative valence group were told that they had to look at one picture they chose for three minutes.

### Analysis

First, we investigated the effects of the control condition, choice, action and stimulus valence on rating change between the two sessions. For this, the rating change from session 1 to 3 was entered as dependent variable in a 2x2x2x2 mixed ANOVA with within subject factors of Choice (Chosen/Unchosen), Condition (Session 1 Choice/Session 2 Choice) and Action (Go/No Go) and a between subject factor of group (positive valence/negative valence). Choice refers to the choice the subjects made for Session 1 Choice pairs before the second rating session and for Session 2 Choice items after the second rating session. The interaction of Choice and Condition revealed whether the rating change differed when the choice preceded the second rating (rate-choose-rate) or did not exceed rating variation due to noise (rate-rate-choose). In other words the interaction term (Choice by Condition) represents the choice-induced preference change. We then investigated whether this choice-induced preference change was modulated by Action (Choice x Condition x Action), Valence (Choice x Condition x Valence) or the interaction of the two (Choice x Condition x Action x Valence). To slice the interaction, the interaction term representing choice-induced value change was analysed with paired t-tests between the Chosen by Go/Chosen by No Go conditions in both valence groups. We also investigated whether the raw choice spread (main effect of Choice) was modulated by Action or Valence (Choice x Action and Choice x Valence). In order to estimate the size of the choice-induced value change without control condition, the rate-choose-rate condition was analysed in a 2x2 within (Action and Choice) x2 between (group) design. See S1 File for the data set and S2 File for additional analyses.

Additionally, our paradigm allowed us to test whether there is an intrinsic choice preference associated with Go or No Go. Pictures are paired with matched ratings and a picture is presented to be chosen by Go or No Go by virtue of a random allocation. Therefore the frequency of subject’s choosing to perform a Go or No Go response should be equal. If the ratio of Go and No Go choices deviates from 1 then this reflects the fact that subjects have an intrinsic preference to express choice by either action or inaction, which may cause deviation from optimal choice. This was assessed with a one sample t-test against 1 for the ratio of Go and No Go responses calculated for each subject. A two sample t-test was used to test whether this bias differs across the two valence groups. To determine whether one group actually rated the pictures lower, a two sample t-test was used to test the mean of all ratings from one group against the other. Trials in which subjects failed to respond were excluded from the analysis. Statistical analysis was carried out with Matlab R2012b and SPSS 19. As the size of the effect of action and valence on choice-induced value change is not known the sample size was determined beforehand based on existing studies on choice-induced preference change and action [10,16,17,21].

## Results

The mean rating of the images given by subjects differed across the two groups and were higher in the positive valence group (95% CIs 4.14–4.83 vs. 3.13–3.7; t(49) = 4.98, p<.001; 95% CI. 64–1.5). Note that this difference occurred even though subjects were instructed to scale their ratings relatively to the stimuli in the set and use the whole range of the scale.

**Fig 2:**
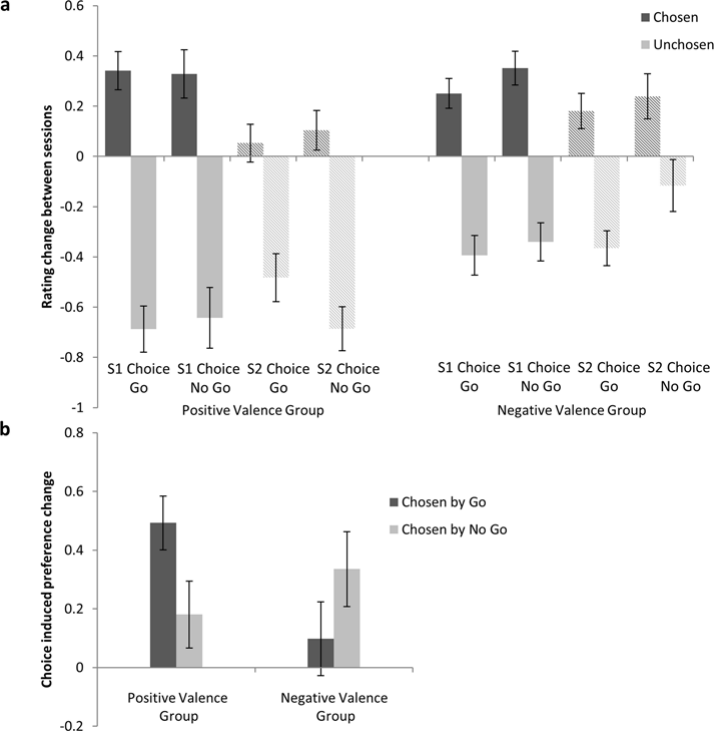
Results. a. The Rating change between the two sessions is displayed for the eight conditions (resulting from the factors Session 1 Choice/Session 2 Choice, Chosen/Unchosen, Go/No Go) in the positive and negative valence group. There was an effect of Choice (chosen larger than unchosen), that was stronger in the experimental condition (larger in Session 1 Choice than Session 2 Choice). In the positive valence group choosing a picture by action led to increased positive choice-induced preference change (defined as the interaction term of Choice and Condition, see Fig. 2b). This effect was absent in the negative valence group. Note that the overall interaction effect is driven by differences across all conditions, including the Session 2 Choice condition. b. To illustrate the overall interaction, the choice-induced preference change (interaction term of Condition (Session 1 Choice/Session 2 Choice) and Choice (Chosen/Unchosen)) is displayed for choice made by action and inaction in both groups, resulting in four conditions (Choice by action in the positive valence group; Choice by inaction in the positive valence group; Choice by action in the negative valence group; Choice by inaction in the negative valence group). Error bars represent the standard error of the mean.

As displayed in Fig. 2a, the value change revealed significant enhancements by an image being chosen by the subject in either the first or second choice session (main effect of Choice F(1,49) = 342.69, p<.001, partial η^2^ = .86). The strong effect of choice is not surprising as it captures both an enhancement by choice, as well as alignment of choice with the drift of the noise contained in the initial measurements [6,7]. The crucial comparison is the difference in value change between the choices that were made before and after the second evaluation. Indeed, we show that a preference change induced by choice is significantly larger if the choice was made in Choice Session 1 (rate-choose-rate) compared to Choice Session 2 (rate-rate-choose; Interaction of Choice and Condition: F(1,49) = 39.28, p<.001, partial η^2^ = .45). This is illustrated by values being larger than 0 in Fig. 2b. Only considering the choice spread in the rate-choose-rate condition alone provides an estimate of by how much one would overestimate the choice-induced preference change without appropriate control condition (F(1,49) = 316.86, p<.001, partial η^2^ = .866).

As displayed in Fig. 2b, for items with positive valence, this choice-induced preference change was stronger when items were selected by choice compared to being selected by passive inaction (t(23) = 2.13, p = .044; 95% CI. 01-.61). This effect differed significantly from the effect in the negative valence stimulus set (F(1,49) = 5.82, p = .02, partial η^*2*^ = .11), which went in the opposite direction (No Go was revaluated insignificantly more positively, t(23) = 1.39, p = .18, 95% CI-.59-.12). As visible in Fig. 2a, the interaction effects involving action and valence are driven by changes across all conditions, including the Session 2 Choice condition.

Additionally, stimulus valence (group) affected the aforementioned choice spread (interaction of Choice and Valence: F(1,49) = 13.11, p = .001, partial η^2^ = .211), yet not the genuine choice induced-preference change (Interaction of Choice, Condition and Valence interaction: F(1,49) = 1.84, p = .18, partial η^2^ = .036). As visible in Fig. 2a, the tie between choice and value change (including choice following value change, as in the rate-rate-choose condition) is increased when stimuli have positive valence.

Analysing frequency of choice itself showed that subjects were biased towards choices obtained by Go (mean of the ratio of Go/No Go responses was 1.13+-.38, the size of the bias (deviation of the choice-ratio from 1) is significantly larger than zero; t(50) = 2.83, p = .007; 95% CI. 039-.23) and notably this Go bias did not differ across the valence groups (t(49) = .41, p = .69; 95% CI-.15-.23).

## Discussion

The current study measured choice spread in value change in two conditions; before and after a choice was made between stimuli. We found that choosing between two images before revaluation resulted in a larger relative increase in valuation for the chosen option than making the choice after the revaluation. This replicated findings showing that a significant portion of choice-induced preference change is not a methodological artefact (see Izuma & Murayama [7] for review).

Consistency of choice and valuation (choice spread independent of whether the choice was made before or after revaluation) was higher when stimuli were of positive valence. This choice consistency is not merely revaluation caused by the choice but also includes conforming the choice to fluctuations in valuation. The fact that this effect was stronger for positive stimuli shows a choice bias that is sensitive to stimulus valence. Additionally, the magnitude of the choice-induced preference change was not modulated by action or valence per se, but by their interaction. This effect was driven by changes across all conditions, including the control condition. Schonberg at al. [14] showed that subsequent choice is biased after as little as eight repetitions of Go responses that coincided with item presentation. In this study we show that when action is integrated into the choice both choice and valuation are affected. Note that action and inaction choice trials happen once per item and merely differ by a single keypress obviating any learning effect per se. A possible explanation for the effects that valence and action have on choice and valuation involves a Pavlovian coupling of response tendencies (e.g. action/inaction) with the valence of the outcome (e.g. choosing among overall positive or overall negative outcomes) [15,22]. This is usually seen to arise out of a consequence that in natural environments action and reward tend to be closely aligned, i.e. approach for reward. Furthermore, a recent study shows that devaluation of stimuli can be induced by stopping actions [23]. The present study shows how this intrinsic coupling of action and valence affects forced choices among similarly valued rewards and the dynamics of revaluation. In this regard the findings add to an emerging picture that action expression in itself is relevant for valuation and behavior [16,17,24–26]. A similar account could also explain the increased consistency of choice and valuation for items of positive valence. The fact that dynamic of revaluation is sensitive to stimulus valence favours a Pavlovian congruence account over more non-specific biases, e.g. attentional focus due to action. However, the factors of action and valence also modulate the spread of value due to choice in the Session 2 Choice condition (see Fig. 2a). Effects in the Session 1 Choice condition could be attributed to a change in revaluation caused by active or inactive choice. Effects in the Session 2 Choice condition could be attributed to a drift in ratings between session that interacts with active and inaction choice tendencies (for example the relatively more positive value change in Session 2 Choice Go Unchosen may be due to the relatively high values of both options biasing the choice towards action). Therefore, the precise effects of action and valence on the dynamics of revaluation, i.e. the interaction of choice biasing valuation and vice versa, remain to be established. A disposition towards viewing the outcomes of active choices more favourably post choice may provide a basis for cognitive biases, e.g. the aforementioned asymmetry in regret over committed or omitted action [27]. A role for action rather than passive inaction may also underpin the omission bias in moral judgements. Here omitting an action that causes harm is seen as more favourable than committing an action with the same outcome. One possibility is this is related to an increased sense of causality and personal responsibility associated with overt actions [28]. Therefore, the effect of action on dynamics of choice-induced preference change is also consistent with accounts of preference change focusing on self-perception (see [13,29] for discussions).

The results also demonstrate how choice can be biased towards action. The fact that choices associated with action are preferred over choices associated with inaction suggests a possible source for suboptimal decision making. Given that this bias is independent of valence it may reflect a general action bias observed in instrumental learning under uncertainty [24,30–32]. This choice bias is especially interesting because it makes explanations of enhanced revaluation due to effort [33,34] less plausible for the effect of action on the dynamics of choice-induced preference change. If the key press was perceived as effortful one would expect subjects to be biased against pressing the key, as item values were closely matched. By contrast, a general bias towards action could be explained by a higher hedonic value of action itself if inaction is perceived as inhibition [35,36].

In conclusion, our findings indicate that actions are not only the expression of a decision process but also impact on both revaluation and choice.

## Supporting Information

S1 File Data file containing the raw data of both experiments. (XLSX)

S2 File Additional descriptive statistics and results from analyses detailed in the main text. (DOCX)

## References

[1] Byrne RMJ. Mental models and counterfactual thoughts about what might have been. Trends Cogn Sci. 2002;6: 426–431. 12413576 10.1016/s1364-6613(02)01974-5

[2] Rajagopal P, Raju S, Unnava HR. Differences in the cognitive accessibility of action and inaction regrets. J Exp Soc Psychol. 2006;42: 302–313.

[3] Kahneman D, Tversky A. The Psychology of Preferences. Sci Am. 1982;246: 160–173.

[4] Ngbala A, Branscombe NR. When does action elicit more regret than inaction and is counterfactual mutation the mediator of this effect? J Exp Soc Psychol 1997;33: 324–343.

[5] Ariely D, Norton MI. How actions create—not just reveal—preferences. Trends Cogn Sci. 2008;12: 13–16. 18063405 10.1016/j.tics.2007.10.008

[6] Chen MK, Risen JL. How Choice Affects and Reflects Preferences: Revisiting the Free-Choice Paradigm. J Pers Soc Psychol. 2010;99: 573–594. 10.1037/a0020217 20658837

[7] Izuma K, Murayama K. Choice-induced preference change in the free-choice paradigm: a critical methodological review. Front Psychol. 2013;4: 41 10.3389/fpsyg.2013.00041 23404185 PMC3566335

[8] Festinger L. A theory of cognitive dissonance. Evanston, Ill.: Row. 291 p. p; 1957.

[9] Sharot T, Fleming SM, Yu X, Koster R, Dolan RJ. Is Choice-Induced Preference Change Long Lasting? Psychol Sci. 2012; 23: 1123–29. 10.1177/0956797612438733 22933456 PMC3802118

[10] Izuma K, Matsumoto M, Murayama K, Samejima K, Sadato N, et al Neural correlates of cognitive dissonance and choice-induced preference change. Proc Natl Acad Sci U S A. 2010:107: 22014–22019. 10.1073/pnas.1011879108 21135218 PMC3009797

[11] Sharot T, Velasquez CM, Dolan RJ. Do decisions shape preference? Evidence from blind choice. Psychol Sci. 2010;21: 1231–1235. 10.1177/0956797610379235 20679522 PMC3196841

[12] Nakamura K, Kawabata H. I choose, therefore I like: preference for faces induced by arbitrary choice. PLoS One. 2013;8: e72071 10.1371/journal.pone.0072071 23977211 PMC3745416

[13] Johansson P, Hall L, Tarning B, Sikstrom S, Chater N. Choice Blindness and Preference Change: You Will Like This Paper Better If You (Believe You) Chose to Read It! J Behav Decis Making. 2014;27: 281–289.

[14] Schonberg T, Bakkour A, Hover AM, Mumford JA, Nagar L, Perez J, et al Changing value through cued approach: an automatic mechanism of behavior change. Nat Neurosci. 2014;17: 625–630. 10.1038/nn.3673 24609465 PMC4041518

[15] Guitart-Masip M, Duzel E, Dolan R, Dayan P. Action versus valence in decision making. Trends Cogn Sci. 2014;18: 194–202. 10.1016/j.tics.2014.01.003 24581556 PMC3989998

[16] Guitart-Masip M, Fuentemilla L, Bach DR, Huys QJ, Dayan P, Dolan RJ, et al Action dominates valence in anticipatory representations in the human striatum and dopaminergic midbrain. J Neurosci. 2011; 31: 7867–7875. 10.1523/JNEUROSCI.6376-10.2011 21613500 PMC3109549

[17] Guitart-Masip M, Chowdhury R, Sharot T, Dayan P, Duzel E, Dolan RJ. Action controls dopaminergic enhancement of reward representations. Proc Natl Acad Sci U S A. 2012;109: 7511–7516. 10.1073/pnas.1202229109 22529363 PMC3358848

[18] Sharot T, De Martino B, Dolan RJ. How choice reveals and shapes expected hedonic outcome. J Neurosci. 2009;29: 3760–3765. 10.1523/JNEUROSCI.4972-08.2009 19321772 PMC2675705

[19] Brehm JW. Postdecision Changes in the Desirability of Alternatives. J Abnorm Psychol. 1956;52: 384–389. 13318848 10.1037/h0041006

[20] Lang PJ, Bradley MM, Cuthbert BN. International affective picture system (IAPS): Affective ratings of pictures and instruction manual. Technical Report A-8 University of Florida, Gainesville, FL. 2008.

[21] Sharot T, Shiner T, Brown AC, Fan J, Dolan RJ. Dopamine enhances expectation of pleasure in humans. Curr Biol. 2009;19: 2077–2080. 10.1016/j.cub.2009.10.025 19913423 PMC2801060

[22] Dayan P, Niv Y, Seymour B, Daw ND. The misbehavior of value and the discipline of the will. Neural Netw. 2006;19: 1153–1160. 16938432 10.1016/j.neunet.2006.03.002

[23] Wessel JR, O’Doherty JP, Berkebile MM, Linderman D, Aron AR. Stimulus Devaluation Induced by Stopping Action. J Exp Psychol Gen. 2014; 143(6): 2316–2329. 10.1037/xge0000022 25313953 PMC4244281

[24] Guitart-Masip M, Huys QJ, Fuentemilla L, Dayan P, Duzel E, Dolan RJ. Go and no-go learning in reward and punishment: interactions between affect and effect. Neuroimage. 2012;62: 154–166. 10.1016/j.neuroimage.2012.04.024 22548809 PMC3387384

[25] Levita L, Hoskin R, Champi S. Avoidance of harm and anxiety: a role for the nucleus accumbens. Neuroimage. 2012;62: 189–198. 10.1016/j.neuroimage.2012.04.059 22569544

[26] Crockett MJ, Clark L, Robbins TW. Reconciling the role of serotonin in behavioral inhibition and aversion: acute tryptophan depletion abolishes punishment-induced inhibition in humans. J Neurosci. 2009;29: 11993–11999. 10.1523/JNEUROSCI.2513-09.2009 19776285 PMC2775933

[27] Gilovich T, Medvec VH, Kahneman D. Varieties of regret: A debate and partial resolution. Psychol Rev. 1998;105: 602–605.

[28] Spranca M, Minsk E, Baron J. Omission and Commission in Judgment and Choice. J Exp Soc Psychol. 1991;27: 76–105.

[29] Aronson E. Dissonance, hypocrisy, and the self-concept In. Harmon-Jones E, Mills J, editor. Cognitive Dissonance: Progress on a Pivotal Theory in Social Psychology. Washington DC: American Psychological Association; 1999 pp. 103–126.

[30] Guitart-Masip M, Economides M, Huys QJ, Frank MJ, Chowdhury R, Duzel E, et al Differential, but not opponent, effects of L-DOPA and citalopram on action learning with reward and punishment. Psychopharmacology 2014; 231: 955–966. 24232442 10.1007/s00213-013-3313-4 PMC3923110

[31] Chowdhury R, Guitart-Masip M, Lambert C, Dolan RJ, Duzel E. Structural integrity of the substantia nigra and subthalamic nucleus predicts flexibility of instrumental learning in older-age individuals. Neurobiol Aging. 2013; 34: 2261–2270. 10.1016/j.neurobiolaging.2013.03.030 23623600 PMC3713434

[32] Cavanagh JF, Eisenberg I, Guitart-Masip M, Huys Q, Frank MJ. Frontal theta overrides pavlovian learning biases. J Neurosci. 2013; 33: 8541–8548. 10.1523/JNEUROSCI.5754-12.2013 23658191 PMC3707146

[33] Zentall TR. Justification of Effort by Humans and Pigeons: Cognitive Dissonance or Contrast? Curr Dir Psychol Sci. 2010;19: 296–300.

[34] Norton MI, Mochon D, Ariely D. The IKEA effect: When labor leads to love. J Consum Psychol. 2012;22: 453–460.

[35] Parkinson J, Haggard P. Hedonic value of intentional action provides reinforcement for voluntary generation but not voluntary inhibition of action. Conscious Cogn. 2013;22: 1253–1261. 10.1016/j.concog.2013.08.009 24021853

[36] Brass M, Haggard P. To do or not to do: the neural signature of self-control. J Neurosci. 2007;27: 9141–9145. 17715350 10.1523/JNEUROSCI.0924-07.2007 PMC6672190

