# Supplementary material for: Action and Valence Modulate Choice and Choice-Induced Preference Change
Source: PLoS One. 2015 Mar 6;10(3):e0119682. doi: 10.1371/journal.pone.0119682 (PMC4352030; doi:10.1371/journal.pone.0119682)
Supplement: S2 File — (DOCX) [file pone.0119682.s002.docx]

**Supplementary Information**

| **Condition** | **Mean** | **Std. Dev** |
| --- | --- | --- |
| **Positive Valence Group** |  |  |
| Experimental (RCR) Go Chosen | .3411 | .37218 |
| Experimental (RCR) Go Unchosen | -.6870 | .45080 |
| Experimental (RCR) No Go Chosen | .3284 | .47244 |
| Experimental (RCR) No Go Unchosen | -.6421 | .59030 |
| Control (RRC) Go Chosen | .0532 | .36768 |
| Control (RRC) Go Unchosen | -.4821 | .46783 |
| Control (RRC) No Go Chosen | .1043 | .38867 |
| Control (RRC) No Go Unchosen | -.6855 | .42699 |
| **Negative Valence Group** |  |  |
| Experimental (CRC) Go Chosen | .2507 | .30945 |
| Experimental (CRC) Go Unchosen | -.3934 | .40894 |
| Experimental (CRC) No Go Chosen | .3510 | .34922 |
| Experimental (CRC) No Go Unchosen | -.3398 | .39430 |
| Control (RRC) Go Chosen | .1809 | .36333 |
| Control (RRC) Go Unchosen | -.3651 | .35992 |
| Control (RRC) No Go Chosen | .2392 | .46340 |
| Control (RRC) No Go Unchosen | -.1160 | .53749 |

| **2x2x2 Within x2 Between** | | | |
| --- | --- | --- | --- |
| **Factor** | **F(1,49)** | **p** | **Partial Eta Squared** |
| Condition | .855 | .36 | .017 |
| Condition* Group | 1.94 | .17 | .038 |
| Action | 1.46 | .23 | .029 |
| Action * Group | 4.23 | .045 | .079 |
| Choice | 342.69 | <.001 | .875 |
| Choice * Group | 13.11 | .001 | .211 |
| Condition * Action | .01 | .92 | <.001 |
| Condition * Action * Group | 1.2 | .28 | .024 |
| Condition * Choice | 39.28 | <.001 | .445 |
| Condition * Choice * Group | 1.84 | .18 | .036 |
| Action * Choice | .08 | .78 | .002 |
| Action * Choice * Group | 3.16 | .08 | .061 |
| Condition * Action *Choice | .11 | .75 | .002 |
| Condition * Action * Choice * Group | 5.816 | .02 | .106 |

| **2x2x2 Within Positive Valence Group** | | | |
| --- | --- | --- | --- |
| **Factor** | **F(1,23)** | **p** | **Partial Eta Squared** |
| Condition | 5.42 | .029 | .191 |
| Action | .48 | .498 | .02 |
| Choice | 227.03 | <.001 | .908 |
| Condition * Action | .89 | .356 | .037 |
| Condition * Choice | 30.95 | <.001 | .574 |
| Action * Choice | 2.1 | .162 | .083 |
| Condition * Action *Choice | 4.55 | .044 | .165 |

| **Within Positive Valence Group – Collapsed over Choice condition** | | | |
| --- | --- | --- | --- |
| **Condition 1 vs** | **Condition 2** | **t(23)** | **p** |
| Experimental (RCR) Chosen by Go | Experimental (RCR) Chosen by No Go | .66 | .518 |
| Experimental (RCR) Chosen by Go | Control (RRC) Chosen by Go | 6.24 | <.001 |
| Experimental (RCR) Chosen by Go | Control (RRC) Chosen by No Go | 2.81 | .01 |
| Experimental (RCR) Chosen by No Go | Control (RRC) Chosen by Go | 4.49 | <.001 |
| Experimental (RCR) Chosen by No Go | Control (RRC) Chosen by No Go | 1.67 | .11 |
| Control (RRC) Chosen by Go | Control (RRC) Chosen by No Go | 2.3 | .031 |

| **2x2x2 Within Positive Valence Group** | | | |
| --- | --- | --- | --- |
| **Factor** | **F(1,26)** | **p** | **Partial Eta Squared** |
| Condition | .079 | .781 | .003 |
| Action | 4.52 | .043 | .148 |
| Choice | 119.8 | <.001 | .822 |
| Condition * Action | .44 | .515 | .016 |
| Condition * Choice | 11.64 | .002 | .309 |
| Action * Choice | 1.15 | .293 | .042 |
| Condition * Action *Choice | 1.93 | .177 | .069 |

| **Within Negative Valence Group – Collapsed over Choice condition** | | | |
| --- | --- | --- | --- |
| **Condition 1 vs** | **Condition 2** | **t(26)** | **p** |
| Experimental (RCR) Chosen by Go | Experimental (RCR) Chosen by No Go | .8 | .432 |
| Experimental (RCR) Chosen by Go | Control (RRC) Chosen by Go | .98 | .338 |
| Experimental (RCR) Chosen by Go | Control (RRC) Chosen by No Go | 3.27 | .003 |
| Experimental (RCR) Chosen by No Go | Control (RRC) Chosen by Go | 1.5 | .145 |
| Experimental (RCR) Chosen by No Go | Control (RRC) Chosen by No Go | 2.99 | .006 |
| Control (RRC) Chosen by Go | Control (RRC) Chosen by No Go | 1.34 | .191 |
